# Trends in healthcare expenditure in United States adults with chronic kidney disease: 2002–2011

**DOI:** 10.1186/s12913-017-2303-3

**Published:** 2017-05-22

**Authors:** Mukoso N. Ozieh, Kinfe G. Bishu, Clara E. Dismuke, Leonard E. Egede

**Affiliations:** 1 Division of Nephrology, Medical University of South Carolina, Charleston, SC USA; 2 Center for Health Disparities Research, Division of General Internal Medicine, Medical University of South Carolina, Charleston, SC USA; 3 Health Equity and Rural Outreach Innovation Center, Ralph H. Johnson Department of Veterans Affairs Medical Center, Charleston, SC USA; 4 Center for Patient Care and Outcomes Research (PCOR), Medical College of Wisconsin, 8701 Watertown Plank Road, Milwaukee, WI 53226 USA; 5 Division of General Internal Medicine, Medical College of Wisconsin, Milwaukee, WI USA

**Keywords:** CKD, Healthcare expenditures, Medical expenditure panel survey

## Abstract

**Background:**

This study examines trends in healthcare expenditure in adults with chronic kidney disease (CKD) and other kidney diseases (OKD) in the U.S. from 2002 to 2011.

**Methods:**

One hundred and eighty-seven thousand, three hundred and fourty-one adults aged ≥18 from the Medical Expenditure Panel Survey (MEPS) Household Component were analyzed. CKD and OKD were based on ICD-9 or CCC codes. A novel two-part model was used to estimate the likelihood of any healthcare use and total expenditures. Covariates included individual demographics and comorbidities.

**Results:**

Approximately 711 adults surveyed from 2002 to 2011 had CKD and 3693 had OKD. CKD was more likely among Non-Hispanic Blacks (NHB), Midwest and Western residents while OKD was more likely among Non-Hispanic Whites (NHW), Hispanics, married and Northeast residents. Both CKD and OKD were more likely in ≥45 years, males, widowed/divorced/single, ≤high school educated, publicly insured, Southern residents, poor and low income individuals. All comorbidities were more likely among people with CKD and OKD. Unadjusted analysis for mean expenditures for CKD and OKD vs. no kidney disease was $39,873 and $13,247 vs. $5411 for the pooled sample. After adjusting for covariates as well as time, individuals with CKD had $17,472 and OKD $5014 higher expenditures, while adjusted mean expenditures increased by $293 to $658 compared to the reference year group. Unadjusted yearly expenditures for CKD and OKD in the US population were approximately $24.6 and $48.1 billion, while adjusted expenditures were approximately $10.7 and $18.2 billion respectively.

**Conclusion:**

CKD and OKD are significant cost-drivers and impose a profound economic burden to the US population.

## Background

Chronic kidney disease (CKD) is defined as decreased glomerular filtration rate <60 ml/min/1.73 m2 or presence of one or more markers of kidney damage for >3 months [1]. CKD affects more than 10% of United States adults [2]. It is the 18^th^ leading cause of death globally [3], an 82% increase in absolute number of deaths in two decades. Unfortunately, the majority of patients with CKD, especially early-stage, are unrecognized so there is a huge propensity for delayed diagnosis and progression to End Stage Renal Disease (ESRD) [4, 5].

CKD is an expensive disease [6] and a public health burden [7, 8]. Recognized non-dialysis CKD patients account for 18.2% of total Medicare expenditures, which is approximately $45.5 billion [9]. CKD patients incur approximately $22,348/person/year in medical expenditures, which is almost three times as much as non-CKD patients [9]. ESRD on the other hand, costs about $34.3 billion [9], with an annual growth of 6–12% [10].

Studies have examined the cost of CKD in the Medicare population and in a managed care setting [9, 11]. However, there are no nationally representative studies on trends in the direct cost of CKD in the US population. This study examines the trend in healthcare expenditures in US adults with CKD and other kidney diseases (OKD) over a 10-year period using a novel cost estimation methodology and a nationally representative survey. The financial burden of CKD is quantified for the US population from 2002 to 2011.

## Methods

### Sample

The Medical Expenditure Panel Survey Household Component (MEPS-HC) data from 2002 to 2011 for individuals aged ≥18 was used for this retrospective study. MEPS-HC is a nationally representative survey maintained and co-sponsored by the Agency for Healthcare Research and Quality (AHRQ) [12]. It has three components: the Household Component (HC), the Medical Provider Component (MPC) and the Insurance Component (IC) [12]. The household component collects detailed information on sociodemographic characteristics, health conditions, healthcare use and expenditures, sources of payment and health insurance coverage [13]. Information on the HC is collected by self-report, and the MPC requests data on medical and financial characteristics from hospitals, physicians, home health providers, and pharmacies in order to validate and supplement information received from the MEPS-HC respondents [13]. Diagnoses coded according to International Classification of Disease, Ninth Revision, Clinical Modification (ICD-9-CM) are also collected as part of the MPC. Kidney disease related medical conditions and procedures reported by respondents were recorded by an interviewer as verbatim and then converted by professional coders to ICD-9-CM codes. The error rate for coders did not exceed 2.5%. Confidentiality of respondents was protected by collapsing fully specified ICD-9-CM codes into 3 digits [13].

Individuals with CKD were identified from the MEPS-HC medical condition files with ICD-9 codes 585 (chronic renal failure) or 586 (renal failure nos) while CCC codes 156 (nephritis, nephrosis, renal sclerosis), 157 (acute and unspecified renal failure), 160 (calculus or urinary tract) and 161 (other diseases of kidney and ureters) were used to identify individuals with other kidney diseases (OKD). The CCCs were generated using Clinical Classification Software [14], which aggregates the ICD-9-CM conditions, and V-codes of each individual into 260 mutually exclusive clinically homogeneous categories [15].

We merged data from the HC survey of the medical condition files and the full-year consolidated files for each year using the unique person identifier (DUPERSID) in a one-to-one match. We pooled 10-year data to ensure sufficient sample size and increase precision of our estimates. This resulted in an unweighted adult sample of 187,341 individuals (representing a population of 188,708,194 individuals). The design of the MEPS survey includes 5 rounds of interviews covering two full calendar years, and provides data for examining person level changes in selected variables such as expenditures and health status [16]. Since MEPS is an overlapping panel survey, many individuals are in the sample for two consecutive years; thus, samples from year to year are not completely independent and observations are not unique.

The survey has a complex design, which includes clustering, stratification, multistage and disproportionate sampling with oversampling of ethnic minorities [15, 17]. The 10-year data has a common variance structure necessary to ensure compatibility and comparability of variables within the sample design. We adjusted the analytic sampling weight variable by dividing it by the number of years being pooled. The sum of these adjusted weights represents the average annual population size for the pooled period. Thus, our study accounts for the sampling weights, clustering and stratification design, to estimate the nationally representative aggregate and incremental healthcare expenditures for the population. The 2002–2011 direct medical expenditures were adjusted to a common 2014-dollar value using the consumer price index (CPI) obtained from the Bureau of Labor Statistics (BLS) [http://data.bls.gov/cgi-bin/cpicalc.pl].

### Ethics and consent

This study was based on MEPS data (see sample section above), which is publicly available dataset. The authors did not require direct contact with survey participants.

### Availability of data and materials

MEPS is a nationally representative survey maintained and co-sponsored by the Agency for Healthcare Research and Quality (AHRQ).

## Measures

### Variables of Interest

The dependent variable was total direct healthcare expenditure for the calendar year for each individual. Expenditures in MEPS-HC were defined as the sum of direct payments for care provided during the year, including out-of-pocket payments and payments by private insurance, Medicaid, Medicare and other sources, but excludes over-the-counter medications, payments for alternative care services, and indirect payments not related to specific medical events [16]. Medical expenditures were composed of office-based medical provider, hospital outpatient, emergency room, inpatient hospital (including zero night stays), prescription medicine, home health care and other medical expenses (vision aids, medical supplies and equipment). Further details about the medical expenditure methodology are provided in MEPS-HC Appendix 1 [16]. The primary independent variables were CKD and OKD as defined previously.

### Covariates

All covariates used for our analyses were based on self-report. Binary indicators of comorbidities were based on a positive response to the question “Have you ever been diagnosed with diabetes, hypertension, stroke, emphysema, joint pain, arthritis or asthma?” Cardiovascular disease (CVD) represents a positive response to a question “Have you ever been diagnosed with coronary heart disease or angina or myocardial infarction or other heart diseases?” Race/ethnic groups were categorized into four: Non-Hispanic Whites (NHW), Non-Hispanic Blacks (NHB), Hispanic and others. Education was categorized as less than high school (≤ grade 11), high school and college or more. Marital status was coded as married, widowed/divorced/separated, and never married. Gender was coded as female vs. male and age was coded as 18–44, 45–64 and ≥ 65 years. Census region was coded as Northeast, Midwest, South and West. Metropolitan Statistical Area (MSA) was coded yes vs. no as of the end of the year (31st December). Henceforth, we refer to MSA as urban and rural. Health insurance was categorized as private, public only and uninsured at all time(s) in the year. The income level was defined as a percentage of the poverty level and grouped in to four categories: poor (<125%), low income (125% to <200%), middle income (200% to <400%) and high income (≥400%). Calendar year was grouped into five consecutive categories: 2002/03, 2004/05, 2006/07, 2008/09, and 2010/11 for the pooled data.

### Analyses

We estimated any healthcare expenditure and direct medical expenditures with a two-part model [18], which allows for mixed discrete-continuous dependent variables [19]. In the first part, a probit model was estimated for the probability of observing a zero versus positive medical expenditure, and then conditional on having a positive medical expenditure, a generalized linear model (GLM) was estimated. This model has been widely employed in situations where due to a large number of non-users of health services, there are excess zeros in resource use or cost data and the assumption of normality of the error term is not satisfied. In the second part, we used GLM to address the positive skewedness of the dependent variable [19]. The GLM address the positive skewness of the total medical expenditure (dependent variable); however, the total medical expenditure had a high concentration of observations with zero total medical expenditure. To improve the precision of the estimates, we used the two-part model [19, 20]. The GLM also has an advantage over log OLS since it relaxes the normality and homoscedasticity assumptions and avoids bias associated with retransforming to the raw scale. The novel two-part model allows users to leverage the capabilities of calculating marginal effects and their standard errors from the combined parts of the model [19].

All estimates were weighted to represent the civilian non-institutionalized population. Standard errors were corrected to account for the complex design of MEPS with Taylor series linearization of the variance [19]. According to recommendations from the statistics literature, our standard errors for MEPS estimates were based on positive values for the person weight [17]. The weighted model was used to estimate the association of direct medical expenditures with CKD and OKD and to estimate the incremental medical expenditures for individuals with CKD and OKD for the population. To control for confounding, sociodemographic factors including age, sex, race, marital status, education, health insurance, rural/urban residence, region, income level and comorbidities were included in the model.

A modified Park test (MPT), taking into account the complex survey design, was used to determine the appropriate family distribution for the GLM prior to conducting the two-part regression model. This test verified that using a gamma distribution with a log link was the best–fitting GLM model to get consistent estimation of coefficients and marginal effects of medical expenditures. Multicollinearity was checked for predictors of the model taking into account the survey design. The variance inflation factor (VIF) for all predictors used in the model was found to be <1.8, indicating no multicollinearity problems. The F-test for the two-part regression model was significant, which indicated the overall significance of the model. All analyses were performed at the person-level using STATA 13.

## Results

The analyses included 187,341 adults surveyed from 2002 to 2011. Approximately 711 had diagnosed CKD and 3693 had diagnosed OKD. As shown in Table 1, significant differences were observed by kidney disease status. CKD was more likely among NHB, Midwest and Western residents, while OKD was more likely in NHW, Hispanics, married and Northeast residents. Both CKD and OKD were more likely in ≥45 years, males, widowed/divorced/single, ≤ high school education, publicly insured, Southern residents, and poor and low-income individuals. All comorbidities were more likely among people with CKD and OKD relative to individuals without either disease.

**Table 1:** Sample demographics by kidney disease status among adults in the US from 2002 to 2011

Variables	All (%)	No kidney disease (%)	CKD only (%)	^a^Other kidney diseases (%)	*p*-value
N(n)	188,708,194 (187,341)	184,457,065 (182,937)	617,210 (711)	3,633,919 (3693)	
*Age (yrs)*					
18–44	45.7	46.1	18.6	31.5	<0.001
45–64	35.4	35.4	37.3	36.4	
65–85	18.9	18.5	44.1	32.1	
*Gender*					
Male	45.5	45.4	48.5	51.7	<0.001
Female	54.5	54.6	51.5	48.3	
*Race/ethnicity*					
Non-Hispanic White	72.1	72.1	59.2	76.0	<0.001
Non-Hispanic Black	10.5	10.5	26.9	8.6	
Hispanic	11.3	11.3	9.0	11.5	
Other	6.1	6.1	4.9	3.9	
*Marital status*					
Married	55.5	55.5	45.4	58.9	<0.001
Widow/Div/Single	21.3	21.1	41.8	26.4	
Never married	23.2	23.4	12.8	14.7	
*Education category*					
< HS	17.4	17.3	28.8	20.7	<0.001
HS	30.5	30.4	32.3	33.2	
College or more	52.1	52.3	38.9	46.1	
*Insurance*					
Private	72.1	72.3	53.4	66.2	<0.001
Public	16.4	16.1	43.1	25.6	
Uninsured	11.5	11.6	3.5	8.2	
*Metropolitan statistical status*					
MSA	82.9	82.9	78.8	81.7	0.099
Non-MSA	17.1	17.1	21.2	18.3	
*Census region*					
Northeast	18.7	18.7	15.9	19.2	<0.001
Midwest	22.8	22.9	23.4	20.3	
South	35.9	35.8	37.3	41.7	
West	22.6	22.6	23.4	18.8	
*Poverty category*					
Poor/NEA	15.1	15.0	29.9	17.9	<0.001
Low Income	12.9	12.8	20.8	14.6	
Middle Income	30.2	30.2	27.2	28.8	
High Income	41.8	42.0	22.1	38.7	
*Chronic conditions*					
Diabetes	9.5	9.0	52.1	25.1	<0.001
Hypertension	32.9	32.3	87.8	50.9	<0.001
CVD	13.6	13.2	50.6	26.9	<0.001
Stroke	3.5	3.4	18.5	6.7	<0.001
Emphysema	2.1	2.1	4.4	4.0	<0.001
Joint pain	37.9	37.6	59.8	49.6	<0.001
Arthritis	26.1	25.7	53.1	40.0	<0.001
Asthma	10.5	10.4	15.1	10.9	0.037
*Year category*					
Year 2002/03	19.2	19.3	15.1	18.1	0.010
Year 2004/05	19.6	19.6	22.5	17.6	
Year 2006/07	19.9	19.9	18.1	19.5	
Year 2008/09	20.5	20.5	18.5	21.4	
Year 2010/11	20.8	20.7	25.8	23.4	

^a^Other Kidney Diseases includes nephritis, nephrosis, renal sclerosis, other diseases of kidney and ureters, other diseases of bladder and urethra: N is weighted sample size; n is unweighted sample size; % is weighted percentage; Widow/Div/Single is widowed, divorced and separated

**Fig. 1:**
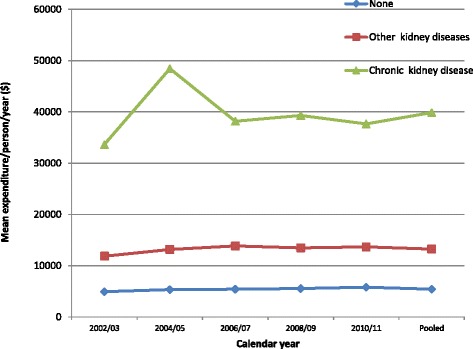
Unadjusted total healthcare expenditures by kidney disease status, 2002–2011

**Fig. 2:**
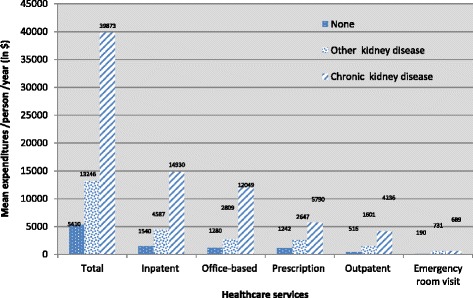
Annual mean expenditures by kidney disease status for the US population, 2002–2011

The mean direct medical expenditures for CKD increased from $33,641 (95% CI $27,129–$40,152) in 2002/2003 to $48,438 (95% CI $35,272–$61,603) in 2004/2005, as shown in Table 2. Mean expenditures then declined to $38,178 (95% CI $30,093–$46,262) in 2006/2007, rose slightly to $39,302 (95% CI $31,641–$46,964) in 2008/2009 and declined again to $37,649 (95% CI $25,531–$49,765) in 2010/2011. Over the decade, individuals with CKD had three and seven times the unadjusted mean expenditures relative to individuals with OKD ($13,247; 95% CI $12,325–$14,169) and no kidney disease ($5411; 95% CI $5305–$5517) respectively (Table 2 and Fig. 1). The main drivers of these differences were inpatient and office-based expenditures (Fig. 2).

**Table 2:** Mean and proportion of total healthcare expenditure by kidney disease status adjusted to 2014 dollars

Expenditure	No kidney disease	CKD only	Other kidney diseases
	Mean (95% CI)	Mean (95% CI)	Mean (95% CI)
2002/03	4927 (4726–5128)	33,641 (27,129–40,152)	11,850 (10,541–13,360)
2004/05	5334 (5088–5581)	48,438 (35,272–61,603)	13,167 (11,612–14,723)
2006/07	5406 (5225–5587)	38,178 (30,093–46,262)	13,853 (11,947–15,759)
2008/09	5537 (5351–5723)	39,302 (31,641–46,964)	13,478 (11,235–15,721)
2010/11	5811 (5596–6027)	37,649 (25,531–49,765)	13,673 (11,517–15,829)
Pooled sample	5411 (5305–5517)	39,873 (34,697–45,049)	13,247 (12,325–14,169)

After adjusting for relevant demographic and comorbidity covariates, individuals with CKD had $17,472 (95% CI $13,785–$21,160) and those with OKD had $5014 (95% CI $4103–$5926) significantly higher incremental expenditures compared to those without kidney disease (Table 3). Other variables that were independently associated with total direct health expenditures were the comorbidities of CVD, stroke, DM and emphysema. The significant demographic factors were being uninsured, publicly insured, age ≥45, female, a racial/ethnic minority, widowed/divorced/single, never married, having a ≥ high school education, an urban dweller, a Southern residence, having a low, middle or high income, and comprising either the 2004/2005, 2006/2007 and 2010/2011 year cohorts. Adjusted mean expenditures increased from approximately $293–$658/year in 2004–2011 relative to the reference year of 2002/03.

**Table 3:** Two-part regression model: Incremental effects of healthcare expenditure by kidney disease status among adults accounting for relevant covariates (adjusted to 2014 dollars)

Variables	Incremental Effect	95% CI	*p*-value
*Primary Independent Variable*			
No Kidney Disease	--	--	--
CKD only	17,472***	13,784–21,160	<0.001
Other Kidney Diseases	5014***	4102–5926	<0.001
*Covariates*			
Age 18–44	--	--	--
Age 45–64	1459***	1211–1707	<0.001
Age 65-85	1946***	1680–2212	<0.001
Male	--	--	--
Female	1123***	904 – 1342	<0.001
NH White	--	--	--
NH Black	−530***	−809 – - 250	<0.001
Hispanic	−1004***	−1267 – -741	<0.001
Others	−959***	−1514 – -405	0.001
Married	--	--	--
Widowed/Divorced/Single	−337***	−542 – -132	0.001
Never married	−490***	−758 – -222	<0.001
Less than high school	--	--	--
High school	489***	199–778	0.001
College or more	780***	515–1054	<0.001
Private	--	--	--
Public insured	966***	633–1298	<0.001
Uninsured	−3083***	−3265–-2901	<0.001
Non-MSA (rural)	--	--	--
MSA (urban)	347**	115–578	0.003
Northeast	--	--	--
Midwest	40	−351–433	0.838
South	−412**	−779–-45	0.027
West	−30	−485–423	0.894
Poor	--	--	--
Low Income	−750***	−1101–-399	<0.001
Middle Income	−1028***	−1356–-700	<0.001
High Income	−830***	−1174–-487	<0.001
Comorbidities (Ref: No disease)	--	--	--
Diabetes	2520***	2246–2794	<0.001
Hypertension	1209***	1014–1405	<0.001
CVD	3384***	3085–3684	<0.001
Stroke	3053***	2553–3549	<0.001
Emphysema	2216***	1702–2731	<0.001
Joint pain	1168***	976–1360	<0.001
Arthritis	1744***	1521–1966	<0.001
Asthma	1497***	1007–1986	<0.001
Year 2002/03	--	--	--
Year 2004/05	476***	162–790	0.003
Year 2006/07	467***	172–762	0.002
Year 2008/09	293**	24–562	0.033
Year 2010/11	658***	346–969	<0.001

** Level of significance p ≤ 0.05, ***level of significance *p* ≤ 0.01; MSA is metropolitan statistical area; Reference for all comorbidities is absence of disease; *Primary outcome variable* in this model is total health expenditures

Based on the average yearly estimate, unadjusted and adjusted total direct medical expenditures for CKD were approximately $24.6 billion and $10.7 billion/year, while OKD were approximately $48.1 billion and $18.2 billion/year in the US population.

## Discussion

We used a nationally representative dataset to analyze the trends in healthcare expenditure in adults with CKD compared to those with OKD and no kidney disease over a 10-year period. This study showed that individuals with CKD had 3 to 7 times the unadjusted expenditures and approximately $17,472 higher adjusted incremental expenditures compared to no kidney disease. The other main drivers of cost in the US include CVD, stroke, diabetes, emphysema, hypertension, arthritis, asthma, joint pain, being age ≥45, female and publicly insured. Compared to 2002/2003, healthcare expenditures from 2004/2005 to 2010/2011 were higher.

Our study has several major contributions to the literature. First, we used a nationally representative dataset to evaluate the per person and U.S. population cost of CKD and OKD over time. Second, this is the first study to examine trends in cost of CKD and OKD using 10-year data. Third, prior analyses have not used our novel methodology, the two-part methodology, to model cost. Fourth, we looked at the incremental effect over time, which allows us to identify how much of the cost is due to CKD and OKD above and beyond baseline cost. Fifth, we were able to estimate the aggregate cost of CKD and OKD on the population using population estimates.

Our analyses revealed a lower prevalence of CKD compared to national estimates [2]. This difference may reflect low CKD awareness in the population and diagnosis by healthcare providers. Studies report low CKD awareness in the US population [21, 22], however our study showed a lower percentage compared to these studies with 2–45%. Methodological differences and our use of administrative codes could be an explanation. A previous report using MEPS data revealed 1.7% of the adult US population reported treatment for kidney disease, similar to our study [23]. We observed higher expenditures in all two-year categories compared to 2002/2003. The most significant change was a 44% increase between 2002/2003 and 2004/2005. We speculate this may be related to an increase in cost of medications (such as erythropoiesis-stimulating agents, intravenous iron and vitamin D etc.), Medicare paid claims, health maintenance organization cost and vascular access expenditures observed in 2004 [24]. Although there was a decrease in clinical service cost for injectables in 2006 [25], this does not completely explain the decline seen in 2006/2007. However, the bundled cost for dialysis treatment implemented in 2011 could explain the decreased expenditure observed in 2010/2011 [26]. The $22,348/person/year cost of CKD according to the USRDS was lower than our $37,649/person/year cost of CKD in 2011 [9]. However, USRDS has a different annual report for patients with End Stage Renal Disease (on dialysis) that exceeds our estimates [9]. Besides differences in methodology and database, MEPS includes only civilian non-institutionalized individuals, all persons with CKD including those on dialysis and comprises all age groups unlike USRDS.

Almost all variables included in our model were independently associated with either a decrease or an increase in total expenditures except residence in the Northeast and West. This implies, although CKD and OKD are important cost-drivers in the US, there are other drivers of cost such as CVD, stroke, DM and emphysema. In relative proportions, CKD was associated with $17,472 and OKD with $5014, while each of these comorbidities were associated with < $4000. In essence, CKD and OKD are significant cost-driver diseases in the US population. The decreased total expenditures associated with uninsured status, minorities and low-middle income individuals could be a reflection of barriers in access to care [27, 28].

This study reinforces the economic and public health burden of CKD and OKD in the US. Given the huge economic burden of CKD, implementation of aggressive strategies by healthcare providers and policymakers to decrease the burden of CKD is of the essence. The high burden of CKD can be addressed in several ways: early recognition/diagnosis, promotion of lifestyle modification, prevention, and aggressive treatment of risk factors for disease progression. The Kidney Disease Outcomes Quality Initiative (KDOQI) guidelines recommend minimization of exposure to nephrotoxins in people with CKD [29]. In addition, studies [30–33] recognize that CKD is a risk factor for acute kidney injury (AKI) and preventable medical errors, and the impact of AKI on CKD progression. Early recognition is ultimately pivotal to all strategies geared towards reduction of the economic burden of CKD and the onus lies on healthcare providers, namely primary care and specialty physicians, nurses and nurse practitioners, pharmacists etc. By putting in place pointers to identify high-risk CKD individuals, the healthcare environment can prevent avoidable exposure to nephrotoxins, which leads to complications and accelerates CKD progression. Furthermore, empowering individuals with CKD with adequate and necessary knowledge about the disease can help avoid expensive outcomes – end stage renal disease (ESRD) and risky interventions. Future studies should examine the impact of the Patient Protection and Affordable Care Act on healthcare expenditures of Americans with kidney disease, pre and post era.

Some of the limitations of our study include 1) our inability to estimate CKD cost by CKD stage/severity (dialysis versus non-dialysis) and lack of laboratory data in MEPS. This is because in MEPS, ICD-9 and procedure codes for CKD such as dialysis are collapsed in order to protect the confidentiality of respondents. 2) We used administrative codes to identify our cohort, which has a low sensitivity [34] and could result in misclassification and likelihood for cost underestimation. 3) The use of self-reported data in MEPS which limits cost estimates and, 4) the small CKD sample size which limits the power of group comparison and limits our ability to estimate CKD cost caused by diseases such as diabetes and hypertension. Nevertheless, MEPS is the only valid national survey that captures the cost of CKD, thus this study provides novel contributions to the existing literature.

## Conclusion

In summary, this nationally representative study on the trends in healthcare expenditure in adults with CKD show CKD is the most important cost-driver in the US population relative to the relevant covariates examined. Uninsured status, racial/ethnic minorities and low to middle income earners have lower total healthcare expenditures which could reflect barriers in access to care which can culminate in delayed access to care, complications and accelerated cost of healthcare for CKD.
